# The worldwide prevalence of burnout syndrome among bank employees: a systematic review and meta-analysis protocol

**DOI:** 10.1186/s13643-021-01833-z

**Published:** 2021-10-30

**Authors:** Sharareh Azedi Tehrani, Abbasali Keshtkar, Akilesh Ramasamy, Majid Fadaei

**Affiliations:** 1 Faculty of Management and Economics, Tehran Science and Research Branch, IAU University, Tehran, Iran; 2 Department of Health Sciences Education Development School of Public Health, Tehran University of Medical Sciences, Tehran, Iran; 3 Department of Dentistry, Jawaharlal Institute of Postgraduate Medical Education and Research (JIPMER), Karaikal, Puducherry, India; 4 Faculty of Economics, Management and Accounting, Yazd University, Yazd, Iran

**Keywords:** Burnout, Prevalence, Maslach burnout inventory (MBI), Banking industry

## Abstract

**Background:**

Burnout is a syndrome caused by chronic stress in the workplace that has not been successfully managed. Although prevalence of burnout is well documented in different fields, little is known about this syndrome in the context of banking work. The objective of this review will be to assess worldwide pooled prevalence of burnout syndrome among bank employees.

**Methods:**

This is a study protocol for a systematic review. We will search the following electronic databases (from their inception onwards): PubMed/MEDLINE, SCOPUS, Web of Science, PsycINFO, ERIC, EBSCOhost, Emerald Insight, and Google Scholar. Grey literature will be identified through searching SCOPUS, Google Scholar, ProQuest databases, and websites of related organizations. We will consider studies that include any type of employee in the banking industry and report extractable prevalence estimates of burnout. Two reviewers will independently screen all citations, full-text articles, and abstract data. The study methodological quality (or bias) will be appraised using an appropriate tool. If feasible, we will conduct random effect meta-analysis of prevalence data. Additional analyses will be conducted to explore the potential sources of heterogeneity (e.g., setting, sex, burnout assessment method, country, and work hours).

**Discussion:**

This systematic review will assess the worldwide prevalence of burnout syndrome among bank employees. The results of this study will be published in a peer-reviewed journal. As it presents an analysis of published literature, the study does not require ethical approval.

**Systematic review registration:**

PROSPERO CRD42020213565

**Supplementary Information:**

The online version contains supplementary material available at (10.1186/s13643-021-01833-z).

## Introduction

The concept of burnout was independently introduced by Herbert Freuden-berger [1] in 1974 and Christina Maslach [2] in 1976 and defined in a variable manner by different authors based on different models. The term was used to describe the psychological state of health care volunteers who were showing such symptoms as emotional depletion and a loss of motivation [3]. Burnout is also defined as the clinical manifestation of occupational stress most frequently encountered in employees who have direct and intensive demanding and emotionally charged relationships with clients receiving services such as customers of banks or retail trade or patients. It may arise when an individual tries to accomplish too much work in too little time as a result of unrealistic deadlines and expectations [4].

The definition of burnout initially most accepted was proposed by Maslach and Jackson (1981), in which it was understood to be a syndrome characterized by emotional exhaustion, depersonalization, and low professional achievement, which frequently occurs among individuals who work in close contact with other people. Emotional exhaustion (EE) refers to the employee’s feeling of mental fatigue that makes him/ her lack the energy to invest and dedicate to his/ her work. Depersonalization (DP) includes the person’s negative behavior towards colleagues and customers, creation of impersonal relationships, and withdrawal, and reduced sense of personal accomplishment (PA) is the reduction of the employee’s efficiency, productivity, and self-efficacy and is likely to result to his/ her resignation [5].

Banking sector is one of the sectors in which most intense stress, interpersonal relationships, and workload are experienced. Indeed, Banking is a business activity of accepting and safeguarding money owned by other individuals and entities, and then lending out this money in order to earn a profit [6]. Each domain of work in banking involves contact with the public in some manner and also associated with financial responsibilities. These activities may directly or indirectly contribute to burnout syndrome among the bank employees.

Statista (2014) reported that the prevalence of burnout experienced by bankers was found to range from 19% to 54% in the Middle East. Job burnout may be the outcome of a combination of individual risk factors and organizational stressors. More specifically, it is considered a negative side effect of the interaction between the individual and his work environment [7].

So, it is necessary to consider the change in the structure of banking activity, which since the 1990s has ceased to be based on bureaucratic tasks and has started requiring that workers meet commercial, profitability, and new customer goals, thus creating increasing anxiety and competitiveness among peers [8].

During the last two decades, most systematic reviews and meta-analyses on burnout syndrome have been published based on observational studies within the professional categories investigated, such as doctors, teachers, and nurses. Nonetheless, in the context of banking work, data on prevalence of burnout syndrome and comorbidity with other psychiatric disorders are still uncertain and no systematic review or meta-analysis was identified about burnout syndrome among bank employees.

To the best of the researchers’ knowledge, only one literature review has been conducted to date on the burnout syndrome in bank employees [8] aimed to investigate the prevalence of burnout syndrome and related factors in bank employees. Fourteen observational studies were identified in this review and findings were grouped into the socio-demographic, personal, organizational, and labor variables related to burnout. Working hours and direct contact with customers were important factors identified in this review. The authors also recommended further studies to enable better understanding of burnout.

The purpose of this systematic review is to synthesize the research published in this field since publication of Maslach Burnout Inventory and to estimate the worldwide pooled prevalence of burnout syndrome in the banking industry using a random effects model due to considerable heterogeneity expected in the data and the multidimensional nature of the condition. This review will identify the knowledge gaps in the literature and provide suggestions for future studies.

## Review questions

The main research question of this review is as follows: What is the worldwide pooled prevalence of burnout syndrome (as defined by MBI [9]) among bank employees? The review also has the following subquestions: (i) What are the characteristics of burnout syndrome among bank employees? (ii) What are the different methods used to assess burnout among bank employees worldwide? (iii) What are the pooled estimates of levels of burnout in the three dimensions of EE, DP, and PA of MBI among bank employees? (iv) What are the common stressors of burnout syndrome among bank employees? (v) What are the important demographic and other factors associated with burnout syndrome? (vi) What are the potential sources of heterogeneity among the studies reporting on burnout?

## Methods

The present protocol has been registered within the PROSPERO database (registration number CRD42020213565) and is being reported in accordance with the reporting guidance provided in the Preferred Reporting Items for Systematic Reviews and Meta-Analyses Protocols (PRISMA-P) statement [10] (see checklist in Additional file 1). The planned review will be conducted according to the Joanna Briggs Institute (JBI) methodology for systematic reviews of prevalence and incidence [11] and will be reported according to the Preferred Reporting Items for Systematic review and Meta-Analysis (PRISMA) 2020 statement [12].

## Inclusion criteria

### Type of participants

This review will consider studies, conducted worldwide, that include any type of employee in the banking industry. Bank employee means any person in any organizational level who (i) is employed by the bank at the time employee’s employment with the bank ends, (ii) was employed by the bank during the last year of employee’s employment with the bank (or during employee’s employment if employed less than a year), or (iii) is employed by the bank during the restricted period. If a study reported multiple domains and includes clearly reported data on banking employees as a subgroup, it will be included.

### Phenomena of interest

This review will consider studies reporting on the point prevalence of burnout measured by any burnout scale. For the purpose of this review, burnout syndrome will be defined based on MBI scale [9]. Burnout was defined by one abnormal score in one or more of the 3 dimensions of the MBI scale (EE, DP, or PA). Severe burnout was defined by the association of high scores of EE and DP and low score of PA. High EE was defined by an EE score ≥ 27. High DP is defined by a score higher than 10. Low PA was defined by a score lower than 33 [9].Quantitative synthesis of prevalence will be done only with studies using MBI. The data related to other Burnout inventories will be summarized and reported separately where available.

### Context

This review will include only studies conducted in the context of the banking sector.

### Type of studies

Any observational study designs including prospective and retrospective cohort studies and cross-sectional studies reporting extractable prevalence estimates of burnout will be included. Studies have to specifically provide a burnout prevalence estimate in bank employees only or the prevalence has to be deducible based on the presented data. Studies do not have to consider burnout the primary outcome of interest for inclusion in this review.

### Date of publication

All the studies published from 01 January 1982, to whose results the researchers have gained access, will be considered for inclusion in this review. The search will be repeated and updated at each stage of the review to keep it current up to 6 months prior to publication. MBI was published in 1981 and is the key source for definition of burnout syndrome as per this review and so only studies published after 1982 are included for this review. If full text is not accessible or retrievable, through any means, it will be excluded.

### Language of publication

There will be no language limitations in this systematic review and meta-analysis. The studies that reach the selection stage after screening (based on their title and abstract) and meet the necessary final-stage inclusion criteria and have their full text available and have been written in a language other than English will be translated by Google Translate and rechecked by official translators and then assessed for the final selection.

### Sampling method and sample size

The use of probability sampling is a basic requirement in prevalence studies and comes in a variety of forms from simple to complex [13]. Sampling should have been conducted by a random method (simple random sampling, systematic random sampling, stratified random sampling and cluster random sampling or a combination of them). The preliminary studies that have used a non-random sampling method (quota sampling, convenient sampling, purposive sampling, self-selection sampling and snowball sampling) or public calls will be also included in this systematic review. The minimum acceptable sample size for the preliminary studies is 30.

## Information sources and search strategy

To achieve the most inclusive search, the search strategy will be based on two components (outcome and population) and include both commercial and non-commercial databases including grey literature. To find keywords related to outcome component, thesaurus systems, including MeSH, the free text method, and the views of experts will be used. Further keywords will be searched using published systematic reviews and peer-reviewed literature. Then, the peer review of search strategy (based on PRISMA-S) [14], including all related keywords and index terms, will be used to identify errors, missing keywords or subject headings, and other issues within the search strategy in consultation with a research librarian.

Also, a pilot search strategy will be developed based on SCOPUS and PubMed sources to ensure sufficient specificity and sensitivity. The final search strategy will be adapted for each included electronic database using Polyglot Search Translator (PST) [15] and Systematic Review Accelerator (SRA) [16], based at the Bond University Institute for Evidence-Based Healthcare.

The search will be updated every 3 months between last search date and current date, throughout the screening, data extraction, and data analysis. In order to reduce the number of duplicates within the results prior to screening, Systematic Review Assistant-Deduplication Module (SRA-DM) [17] in SRA platform will be employed.

Reference lists of all relevant and selected publications will be searched for additional studies that may have not been obtained. The draft search strategy for SCOPUS is provided in additional file 2.

### Electronic database search

To achieve the study objectives, searches will be carried out in the following electronic databases: Scopus, Google Scholar, PubMed/MEDILINE, psycINFO (EBSCOhost), Science Citation Index Expanded (SCI-Expanded) –1900-present (WoS), Social Sciences Citation Index (SSCI) (WoS), Emerging Sources Citation Index (ESCI) (WoS), ERIC (EBSCOhost), and Emerald Insight, without language restriction for studies on the prevalence of symptoms of burnout in bank employees (i.e., excluding non-banking jobs).

### Grey literature

Furthermore, relevant grey literature (e.g., thesis or dissertations and conference papers) will be included by searching in the electronic databases of ProQuest Dissertation and Theses Global (ProQuest), Scopus, and Google Scholar search engine.

### Others

Further search will be made in documents and white papers published by the World Bank, International Labour Organization (ILO), and World Health organization (WHO) for relevant data on burnout syndrome among bank employees and relevant data will be included in the review.

### Contacting the experts

When contacting experts, they will be asked to send any relevant theses or to introduce conferences related to the subject of this systematic review (in addition to the search conducted in the databases).

## Study screening and selection

All studies identified from the literature search will be uploaded into Mendeley. Also, in order to aid the screening and selection process, HubMeta (hubmeta.com), a recent web-based data entry system, will be employed. In the screening stage, title and abstract of each study is assessed by at least two reviewers independently based on a checklist prepared according to the inclusion and exclusion criteria. To be eligible for inclusion in the review, studies need to meet all of the related criteria. When eligibility is unclear, the study will be provisionally included for the full-text review stage to assess eligibility. Then, in the selection stage, at least two of the reviewers will independently review the full text of the studies obtained in the screening stage and determine eligible studies for inclusion to the next stage.

Any disagreement in the above two stages will be resolved by discussion, and if the disagreement is not resolved, the opinion of a third expert will be involved to achieve consensus.

## Assessment of methodological quality

Studies selected for retrieval will be assessed by at least two independent reviewers for methodological validity before inclusion in the review using standardized critical appraisal instruments from the Joanna Briggs Institute – Critical Appraisal Checklist for Studies Reporting Prevalence Data [18]. Any disagreements that will arise between the reviewers will be resolved through discussion, and if the disagreement is not resolved, a third reviewer will be involved to achieve consensus.

## Data extraction

Data will be extracted from studies included in the review independently by the two reviewers, using an adapted version of the JBI extraction form for prevalence studies [11].

The data extraction form will be piloted by at least two of the reviewers on a sample of included studies independently. After due consensus is achieved, the data extraction form will be finalized for data extraction. If, during the data extraction stage, further information is identified which may need extraction and not available in the form, the data form will be updated to include this field after due discussion and consensus of all reviewers. Each of these steps will be recorded in sufficient detail to keep an audit trail.

The data extracted will include specific details about the populations, outcomes, and other characteristics including the following: (i) general information: reviewer ID, revision date, study date, title, author(s), journal, year of publication; (ii) study characteristics: study design, main/secondary objectives, funding of study, population study (total population, specific group population or other), setting/context, sampling method, sample size, country of study, continent, position of employee, type of age data (mean, median…), sex (male, female, both), number/percentage of men/women, method of data analysis; and outcome information: outcome measure (absolute numbers, point prevalence or periodic prevalence),measure of the prevalence (crude or adjusted measure), outcome subvariables, Burnout inventory, prevalence n/N (%), number of burnout cases, EE proportion, DP proportion, LPA proportion, proportion of bank employees with burnout based on position, overall effect, factors associated, findings consistency, summary of findings, all objectives reported, effect direction, mitigation solution, and conflicts of interest.

Any disagreements that arise between the reviewers at piloting or where the reviewer is uncertain on particular study details to extract will be resolved through discussion or with a third reviewer. If required, the authors of the papers will be contacted to request missing or additional data.

## Data synthesis

The intention of this review is to perform a meta-analysis. Therefore, the reliability of pooled quantitative summary estimates will be judged to ensure that conducting a meta-analysis is possible in this study. Prevalence data extracted from the included studies will, where possible (e.g., studies using uniform case definitions, the same measures of outcome, context and approaches), be pooled in a statistical meta-analysis using related software.

Prevalence data will be pooled using random-effect model and presented as numbers and proportions. The prevalence of burnout and 95% confidence interval (CI) will be calculated using the Binomial Exact Method.

When pooling proportions for meta-analysis, a transformation of the data will be required. Logit or Freeman-Tukey double arcsine transformation of the prevalence will be applied. Prevalence estimates will be transformed to logits to improve their statistical properties.

The final pooled logit will be back transformed, resulting in pooled prevalence and 95% confidence intervals [11, 19].

Heterogeneity will be statistically assessed using the standard *X*^2^ test, Tau-squared, and *I*-squared tests.

The following references will be used as the bases for determining the degree of heterogeneity.

- Heterogeneity values of 0–40% will be taken as “perhaps not important”
- Heterogeneity values of 30–60% as “moderate heterogeneity”
- Heterogeneity values of 50–90% as “substantial heterogeneity”

Heterogeneity values of 75—100% will be taken as “considerable heterogeneity.” The level of statistical significance will be set at *p* <0.1 for the *Q*-test [20]. The importance of the observed value of *I*^2^ depends on (i) magnitude and direction of effects and (ii) strength of evidence for heterogeneity (e.g., *P* value from the chi-squared test and a confidence interval for *I*^2^) [21].

However, when statistical pooling is not possible, because of considerable heterogeneity of the reported measures, the forest plot of the multiple studies will be presented; this is useful for displaying how prevalence estimates vary between studies. In addition, data will be presented in narrative form including tables and figures to aid in data presentation wherever appropriate [11, 19].

The potential sources of heterogeneity will be investigated by arranging groups of studies according to potentially relevant characteristics into subgroups and meta-regression analyses will be conducted to assess the factors. In addition, a thematic assessment of all factors in consultation with subject experts will be done to further probe into causes of heterogeneity. In order to deal with publication bias, the first strategy is to perform the most inclusive search in the search stage of the study.

Also, funnel plots will be used to assess potential reporting bias and non-significant study effect. Begg’s test and Egger’s test will also be performed, and significant results (*p* <0.1) shall suggest a publication bias, in which case the “trim and fill” method will be used.

Sensitivity analyses will be performed to explore the impact of individual studies on the overall calculated prevalence estimates. This will be performed by investigating whether dropping or adding primary studies with (say) slightly non-standard burnout definitions will make a difference.

In the case of missing data in the final included studies, attempts will be made to access the authors’ contact data and complete the missing data by corresponding with them. The lack of access to sufficient data (after sending three emails) shall necessarily mean the elimination of that study from the data synthesis process.

If data is available, meta-analyses will be performed on subgroups of studies reporting emotional exhaustion, depersonalization, personal accomplishment, and overall burnout. Analyses will be stratified by work setting, sex, burnout assessment method, country (geographical area of the study), and work hours (whenever available). The analyses will be performed with STATA (version 14.2) or R (version 4.0.2) using appropriate packages.

## Discussion

The main goal of this review is to assess worldwide pooled prevalence of burnout syndrome among bank employees. Indeed, this study will highlight the proportion along with common stressors of burnout syndrome among bank employees. According to the final result of this study, health experts including policy makers and occupational health and safety specialists in banking sector will be able to prioritize policies and conduct awareness programs to improve bank employees’ health. The review results will help researchers, bank managers, and experts to make better decisions in order to reduce the level of burnout among bank employees and improve job performance. The results of the review will be published in peer-reviewed publications and presented to professional conferences in the form of oral or poster presentations. Any amendments made to this protocol during the conduct of the study will be described and reported in the final manuscript.

## Supplementary Information

**Additional file 1** PRISMA-P (Preferred Reporting Items for Systematic review and Meta-Analysis Protocols) 2015 checklist: recommended items to address in a systematic review protocol.

**Additional file 2** The draft search strategy used in Scopus.

## Data Availability

Not applicable

## Abbreviations

MBI: Maslach Burnout Inventory
EE: Emotional exhaustion
DP: Depersonalization
PA: Personal accomplishment
PRISMA: Preferred Reporting Items for Systematic Reviews and Meta-analyses
PRIMA-S: Preferred Reporting Items for Systematic Reviews and Meta-analyses literature search extension
JBI: Joanna Briggs Institute
SRA: Systematic Review Accelerator
PST: Polyglot Search Translator
SRA-DM: Systematic Review Assistant-Deduplication Module.
