# Supplementary material for: The worldwide prevalence of burnout syndrome among bank employees: a systematic review and meta-analysis protocol
Source: Syst Rev. 2021 Oct 30;10:283. doi: 10.1186/s13643-021-01833-z (PMC8557009; doi:10.1186/s13643-021-01833-z)
Supplement: Supplementary file 2 — Additional file 2 The draft search strategy used in Scopus. [file 13643_2021_1833_MOESM2_ESM.docx]

**Additional file (1): The draft search strategy used in Scopus**

| SCOPUS |
| --- |
| Query |
| TITLE-ABS-KEY(burnout) OR TITLE-ABS-KEY("professional burnout") OR TITLE-ABS-KEY("occupational burnout") OR TITLE-ABS-KEY(burnout AND career) OR TITLE-ABS-KEY(overstrain) OR TITLE-ABS-KEY("neurocirculatory asthenia") OR TITLE-ABS-KEY("industrial fatigue") OR TITLE-ABS-KEY("burned out") OR TITLE-ABS-KEY("professional exhaustion syndrome") OR TITLE-ABS-KEY("a feeling of being finished") OR TITLE-ABS-KEY(depersonalization) OR TITLE-ABS-KEY("emotional exhaustion") OR TITLE-ABS-KEY("physical exhaustion") OR TITLE-ABS-KEY("sense of frustration") OR TITLE-ABS-KEY("sense of failure") OR TITLE-ABS-KEY("professional  stressors") OR TITLE-ABS-KEY("emotional stressors") OR TITLE-ABS-KEY("psychological stress") OR TITLE-ABS-KEY("professional stress") OR TITLE-ABS-KEY("psychological stressors") OR TITLE-ABS-KEY(stress) OR TITLE-ABS-KEY("psychological fatigue") OR TITLE-ABS-KEY("emotional stress") OR TITLE-ABS-KEY("compassion fatigue") OR TITLE-ABS-KEY("work fatigue") AND TITLE-ABS-KEY("bank employees") OR TITLE-ABS-KEY(“bank staff”) OR TITLE-ABS-KEY("bank personnel") OR TITLE-ABS-KEY("bank office") OR TITLE-ABS-KEY("banking") OR TITLE-ABS-KEY("bank frontline employee") OR TITLE-ABS-KEY("banking sector") OR TITLE-ABS-KEY(“bank worker”) AND PUBYEAR > 1982 |
